# Investigating cartilage-related diseases by polarization-resolved second harmonic generation (P-SHG) imaging

**DOI:** 10.1063/5.0196676

**Published:** 2024-04-30

**Authors:** Kausalya Neelavara Makkithaya, Nirmal Mazumder, Wei-Hsun Wang, Wei-Liang Chen, Ming-Chi Chen, Ming-Xin Lee, Chin-Yu Lin, Yung-Ju Yeh, Gregory J. Tsay, Sitaram Chopperla, Krishna Kishore Mahato, Fu-Jen Kao, Guan-Yu Zhuo

**Affiliations:** 1 Department of Biophysics, Manipal School of Life Sciences, Manipal Academy of Higher Education, Manipal, Karnataka 576104, India; 2 Institute of Translational Medicine and New Drug Development, China Medical University, Taichung 404328, Taiwan; 3 Center for Condensed Matter Sciences, National Taiwan University, Taipei 10617, Taiwan; 4 Department of Biomedical Sciences and Engineering, Tzu Chi University, Hualien 97004, Taiwan; 5 Autoimmune Disease Laboratory, China Medical University Hospital, Taichung 404327, Taiwan; 6 School of Medicine, China Medical University, Taichung 404328, Taiwan; 7 Division of Immunology and Rheumatology, Department of Internal Medicine, China Medical University Hospital, Taichung 404327, Taiwan; 8 Department of Orthopedics, Kasturba Medical College, Manipal Academy of Higher Education, Manipal, Karnataka 576104, India; 9 Institute of Biophotonics, National Yang Ming Chiao Tung University, Taipei 11221, Taiwan

## Abstract

Establishing quantitative parameters for differentiating between healthy and diseased cartilage tissues by examining collagen fibril degradation patterns facilitates the understanding of tissue characteristics during disease progression. These findings could also complement existing clinical methods used to diagnose cartilage-related diseases. In this study, cartilage samples from normal, osteoarthritis (OA), and rheumatoid arthritis (RA) tissues were prepared and analyzed using polarization-resolved second harmonic generation (P-SHG) imaging and quantitative image texture analysis. The enhanced molecular contrast obtained from this approach is expected to aid in distinguishing between healthy and diseased cartilage tissues. P-SHG image analysis revealed distinct parameters in the cartilage samples, reflecting variations in collagen fibril arrangement and organization across different pathological states. Normal tissues exhibited distinct *χ*_33_/*χ*_31_ values compared with those of OA and RA, indicating collagen type transition and cartilage erosion with chondrocyte swelling, respectively. Compared with those of normal tissues, OA samples demonstrated a higher degree of linear polarization, suggesting increased tissue birefringence due to the deposition of type-I collagen in the extracellular matrix. The distribution of the planar orientation of collagen fibrils revealed a more directional orientation in the OA samples, associated with increased type-I collagen, while the RA samples exhibited a heterogeneous molecular orientation. This study revealed that the imaging technique, the quantitative analysis of the images, and the derived parameters presented in this study could be used as a reference for disease diagnostics, providing a clear understanding of collagen fibril degradation in cartilage.

## I. INTRODUCTION

Understanding articular cartilage intricacies is crucial for improving pharmacological and surgical interventions. Found in synovial joints, this specialized connective tissue absorbs impact and distributes loads. With a multi-layered architecture and zones such as superficial, medium, deep, and calcified layers, the extracellular matrix (ECM) includes key components, such as proteoglycans, elastin, hyaluronan, and type-II collagen, the prevalent structural molecules.^1^ The ability of articular cartilage to handle physical loading is due to the interaction of proteoglycans, water, and type-II collagen.^2^ Interstitial swelling and the ability to resist compressive deformation to the tissue are caused by the interaction of the polyanionic aggregating proteoglycans, cations, and water, while the collagenous framework provides resistance against deformation due to tensile forces. The lack of vascularization of the articular cartilage causes it to have an inconsistent nutrient supply, thus resulting in the limited ability of the cartilage to repair itself when damaged, especially with progressive wear and tear, leading to osteoarthritis (OA), a common and debilitating joint disorder.^3^ Rheumatoid arthritis (RA) is an autoimmune disease affecting cartilage that results in chronic joint damage.^4,5^ Unfortunately, the limited regeneration capacity of cartilage results in irreversible damage from OA, RA, or other injuries, eventually leading to joint dysfunction. Moreover, the phenotypic instability of the chondrocytes that are responsible for the maintenance of hyaline cartilage contributes to fibrotic transformations and increased type-I collagen production, thus leading to a weakened ECM.^6–9^ Pathologists have shown that changes in collagen architecture are strongly linked to cartilage deterioration in OA and stress-related joint injuries.^10^

Currently, the diagnosis of OA or RA involves a combination of radiographic assessments, clinical evaluations, imaging tests, and laboratory tests.^11,12^ Synovial fluid analysis complements blood tests and aids in ruling out other forms of arthritis.^13^ Imaging modalities, such as x-rays, ultrasound, and magnetic resonance imaging (MRI), can assist in visualizing articular damage.^14^ Recognizing the critical treatment window within the first two years of arthritis onset emphasizes the importance of early diagnosis and intervention for both OA and RA.^15^ However, diagnostic challenges arise from symptom heterogeneity, lack of specific tests, and overlap with other conditions, such as psoriatic arthritis or systemic lupus erythematosus. A reliable diagnosis requires the consideration of multiple clinical features and examination results. A technique providing *in situ* molecular information on collagen alterations alongside morphological changes would significantly benefit articular cartilage assessment in research and clinical settings.^16^ Such a technique could enhance diagnostic precision and complement the existing diagnostic methods.

Polarized light microscopy (PLM) is a valuable optical imaging technique for the analysis of optically anisotropic materials and is, thus, used in the study of articular cartilage.^17,18^ Many scientists worldwide have used PLM to examine cartilage composition in OA and RA. However, image resolution and contrast are often limited, as the images are formed by the phase retardation of ordinary and extraordinary light waves passing through birefringent tissues. This limitation makes interpreting structural information in complex or thick biological tissues challenging, thus necessitating sophisticated calibration processes for structural definition of the samples.^19^

To increase the image resolution and capture structural and molecular details, we propose polarization-resolved second harmonic generation (P-SHG) microscopy for investigating articular cartilage in the context of OA and RA. SHG microscopy, with polarimetric measurements, uniquely captures signals from non-centrosymmetric structures, such as collagen,^20^ providing sub-micrometer-level resolution in tissue imaging. The expression of type-II collagen is one of the most essential indicators for assessing the quality of articular cartilage construction in cartilage tissue engineering. P-SHG microscopy allows quantitative analysis of structural variations in articular cartilage, including molecular arrangement and collagen fibril architecture in the ECM, which could aid in the diagnosis of cartilage-related disorders.^21^ Changes in collagen alignment and density can indicate cartilage degeneration, OA, RA, or other cartilage-related disorders. Thus, we postulate that these changes in the collagen alignment of the ECM could be detected by the proposed P-SHG microscopy technique.

In the current digital era, quantitative analysis of medical images is essential for gaining in-depth insights into tissue pathology. Texture analysis, a crucial aspect of image analysis, is vital for tasks such as object detection, quality control, and anomaly detection.^22^ The image texture, which captures variations in the spatial distribution, frequency, and intensities of gray-level values in each pixel, is evaluated through methods such as histograms and two-dimensional co-occurrence matrices. Such computational image analysis has been validated for improved predictive modeling and quantitation of histopathology.^23^

The current study focused on the application of P-SHG microscopy to quantitatively study structural variations in articular cartilage in the context of OA and RA. This is achieved by high-resolution images and susceptibility tensor analysis of the tissue for the associated molecular information, which facilitates a deeper understanding of the diagnosis and the pathogenic mechanism regarding the degradation of collagen in the ECM. Additionally, textural analysis of the images was performed using the gray-level co-occurrence matrix (GLCM) method^24,25^ to enhance the comprehension of structural variations in the cartilage at the molecular level. Multiple structural factors derived from P-SHG microscopy enable the characterization of tissue structures, evaluation of treatment effects, and analysis of different types of OA and arthrofibrosis.

## II. RESULTS

**FIG. 1.**
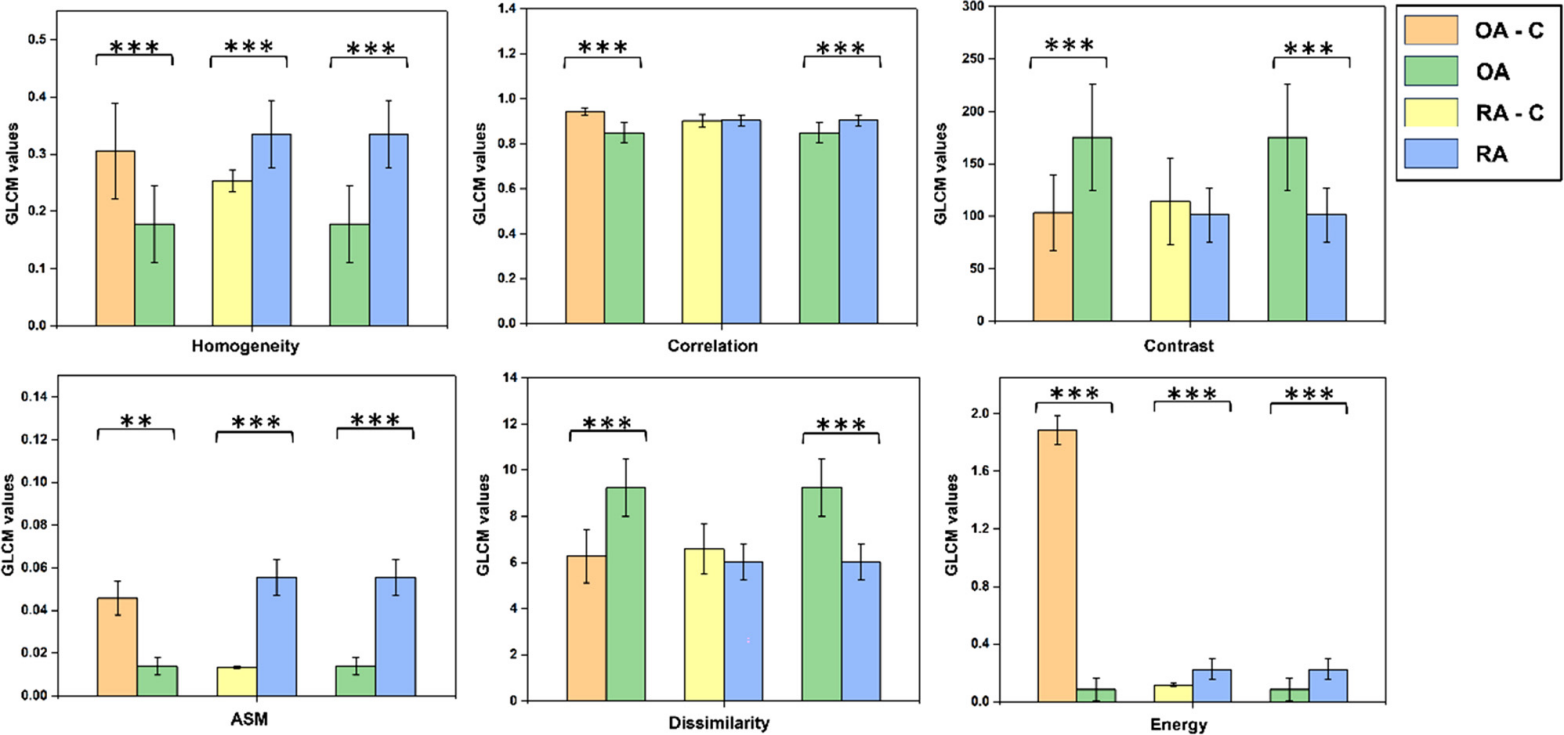
The graphs depict the textural features of the cartilage tissue images. The graphs compare the features extracted from OA-C/OA, RA-C/RA, and OA/RA. ^*^ indicates *p* < 0.05, ^**^ indicates *p* < 0.01, and ^***^ indicates *p* < 0.001. n = 5 for each condition in the analysis.

Distinct tissue arrangements were observed in the control and disease states, prompting an investigation into whether the textural parameters of the GLCM algorithm could distinguish between different pathological states of cartilage tissue. The results of GLCM feature extraction revealed significant differences in textural features among various pathological states of cartilage. Figure 1 illustrates the differences in textural features between cartilage tissue affected by OA and RA and that of the control group. The findings suggest that all textural features of the tissue images exhibit significant differences, indicating the potential of the textural analysis algorithm to detect variations in cartilage surface topology. The control groups, which consisted of normal cartilage samples without any treatment, are abbreviated as OA-C and RA-C for OA and RA, respectively, to provide a basis for comparison.

**FIG. 2.**
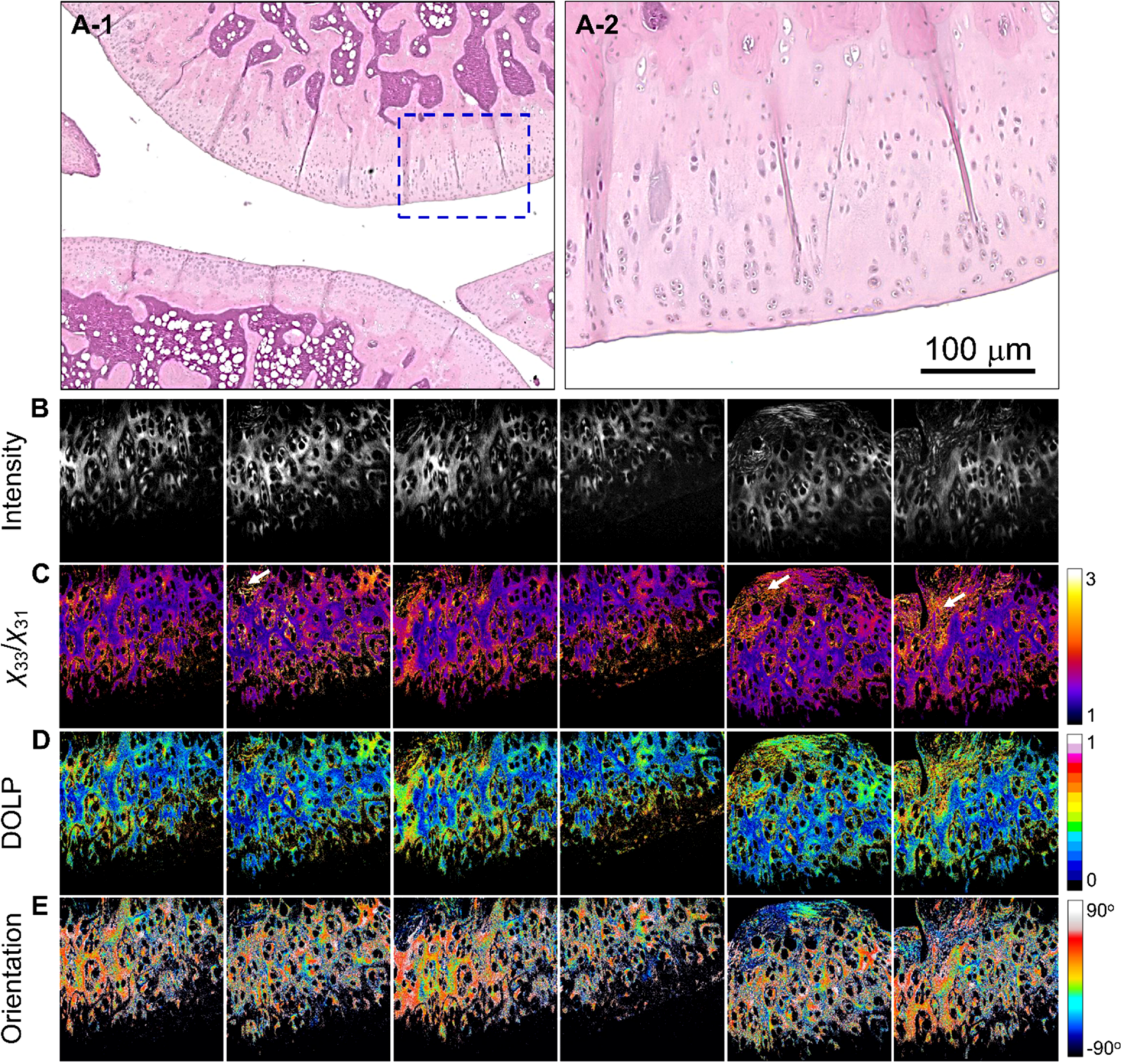
Images of OA-C tissue: (a-1) and (a-2) H&E-stained images, where (a-2) shows a magnified view of the region indicated in (a-1). (b)–(e) are the SHG intensity, *χ*_33_/*χ*_31_, DOLP, and planar orientation of the OA-C tissues, respectively. The color scale shows the values of each parameter. The white arrows indicate the bone tissues in the section. Image size = 400 × 400 *μ*m^2^. Images (b)–(e) are representative and were selected from the samples used (n = 5).

The P-SHG parameter results are presented in Table I, with detailed discussions available in the manuscript. All acquired images and analyses can be found in the supplementary material. Figures 2(a-1) and 2(a-2) show H&E-stained images of the OA-C samples, revealing abundant chondrocytes in the frontal area of the cartilage. The corresponding SHG images [Fig. 2(b)] depict compact type-II collagen in the ECM with chondrocyte edges. The average *χ*_33_/*χ*_31_ value (n = 5) of 1.42 ± 0.195 [Fig. 2(c)] was found to be consistent with the expected values.^2^ Bone tissue (indicated by white arrows) yielding a *χ*_33_/*χ*_31_ value of 1.912 ± 0.439 was excluded from the analysis because it is beyond the scope of the proposed investigation of articular cartilage tissues (see the supplementary material). Cartilage exhibited a lower degree of linear polarization (DOLP) value (0.269 ± 0.121) than did bone (0.510 ± 0.194), indicating that type-I collagen has larger birefringence with a more fibrillar structure [Fig. 2(d)]. The orientation distribution in Fig. 2(e) illustrates diverse collagen fibril orientations, especially around chondrocyte edges.

**TABLE I.** Overall comparison of the P-SHG parameters.

Parameter	Normal cartilage	OA cartilage	RA cartilage
*χ*_33_ */χ*_31_	1.420 ± 0.195 (OA-C)1.537 ± 0.216 (RA-C)	1.861 ± 0.449	1.75 ± 0.337
DOLP	Low	Higher than normal	Higher than normal
Collagen orientation	Wide distribution	More directional	Widespread heterogeneity
Collagen type	Mostly type-II	Increased type-I	Some increase in type-I
Morphology	Intact	Loss of chondrocytes	Erosion and chondrocyte swelling
Degradation mechanism	⋯	Collagen type changes	Collagen erosion/swelling some collagen changes

**FIG. 3.**
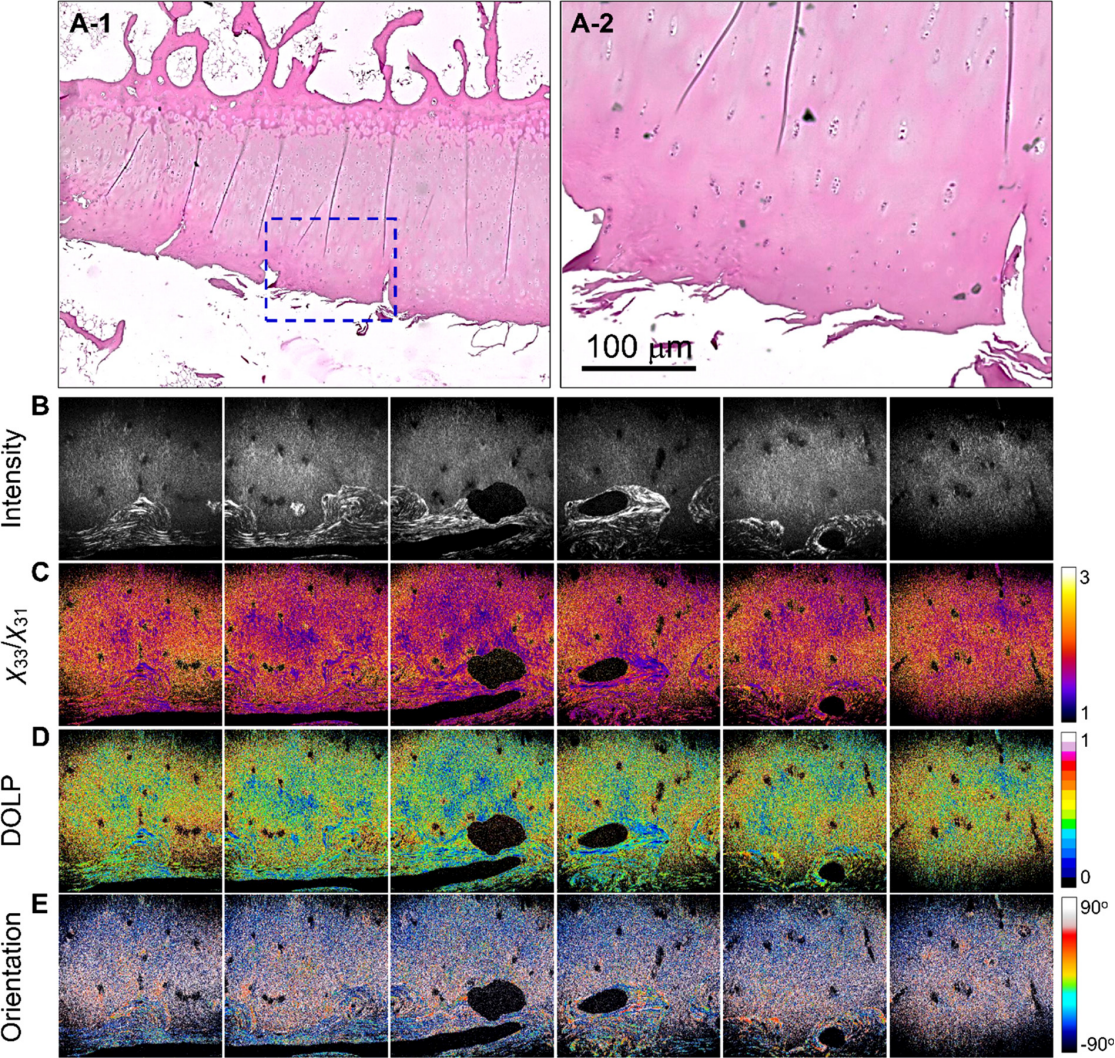
Images of OA tissue: (a-1) and (a-2) H&E-stained images, where (a-2) shows a magnified view of the region indicated in (a-1). (b)–(e) are the SHG intensity, *χ*_33_/*χ*_31_, DOLP, and planar orientation of the OA tissues, respectively. The color scale shows the values of each parameter. Image size = 400 × 400 *μ*m^2^. Images (b)–(e) are representative and were selected from the samples used (n = 5).

Furthermore, H&E-stained images of the OA samples indicated the presence of fewer chondrocytes. Additionally, a reduction in the size of the chondrocytes was observed [Figs. 3(a-1), 3(a-2), and 3(b)].^21^ SHG images revealed a higher *χ*_33_/*χ*_31_ ratio (1.861 ± 0.449, n = 5), suggesting that type-I collagen production is associated with OA pathology [Fig. 3(c)]. The increase in DOLP (0.483 ± 0.216) was also consistent with the increase in type-I collagen [Fig. 3(d)]. The orientation distribution in Fig. 3(e) shows consistency in direction, presenting similar colors in the figure, which is commonly found in the organization of type-I collagen.

**FIG. 4.**
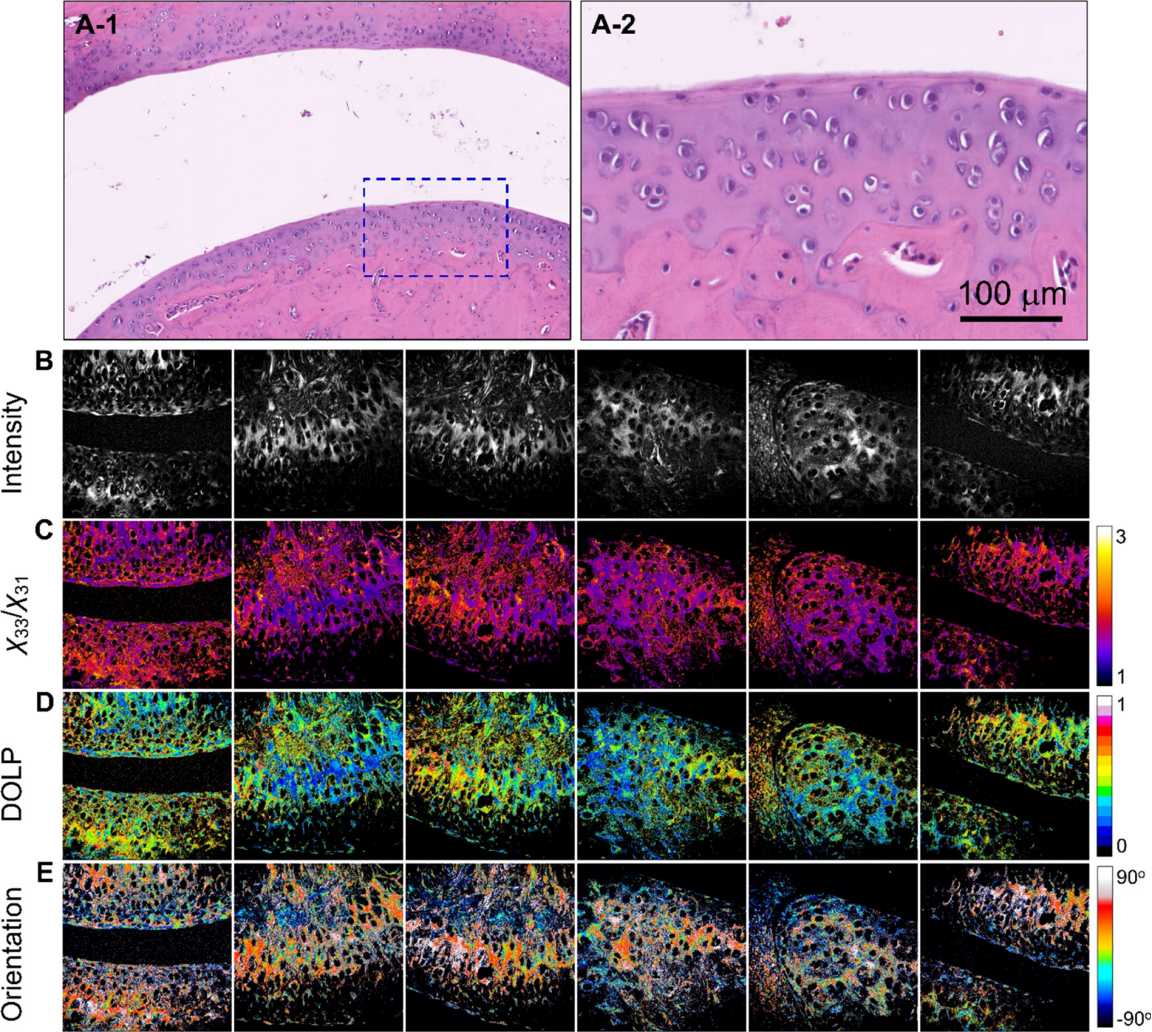
Images of RA-C tissue: (a-1) and (a-2) H&E-stained images, where (a-2) shows a magnified view of the region indicated in (a-1). (b)–(e) are the SHG intensity, *χ*_33_/*χ*_31_, DOLP, and planar orientation of the RA-C tissues, respectively. The color scale shows the values of each parameter. Image size = 400 × 400 *μ*m^2^. Images (b)–(e) are representative and were selected from the samples used (n = 5).

In the RA-C group, the H&E-stained images [Figs. 4(a-1) and 4(a-2)] resemble those of the OA-C group, and the SHG images [Fig. 4(b)] show an average *χ*_33_/*χ*_31_ value (n = 5) of 1.537 ± 0.216 [Fig. 4(c)], akin to that of the OA-C group, indicating a similar organization of collagen in both groups. Additionally, the DOLP value (0.348 ± 0.144) was lower than that of the bone (0.510 ± 0.194) in a manner similar to that of OA-C [Fig. 4(d)]. However, the cartilage exhibited a network of collagen fibrils with a diverse orientation distribution [Fig. 4(e)], resembling that of OA-C.

**FIG. 5.**
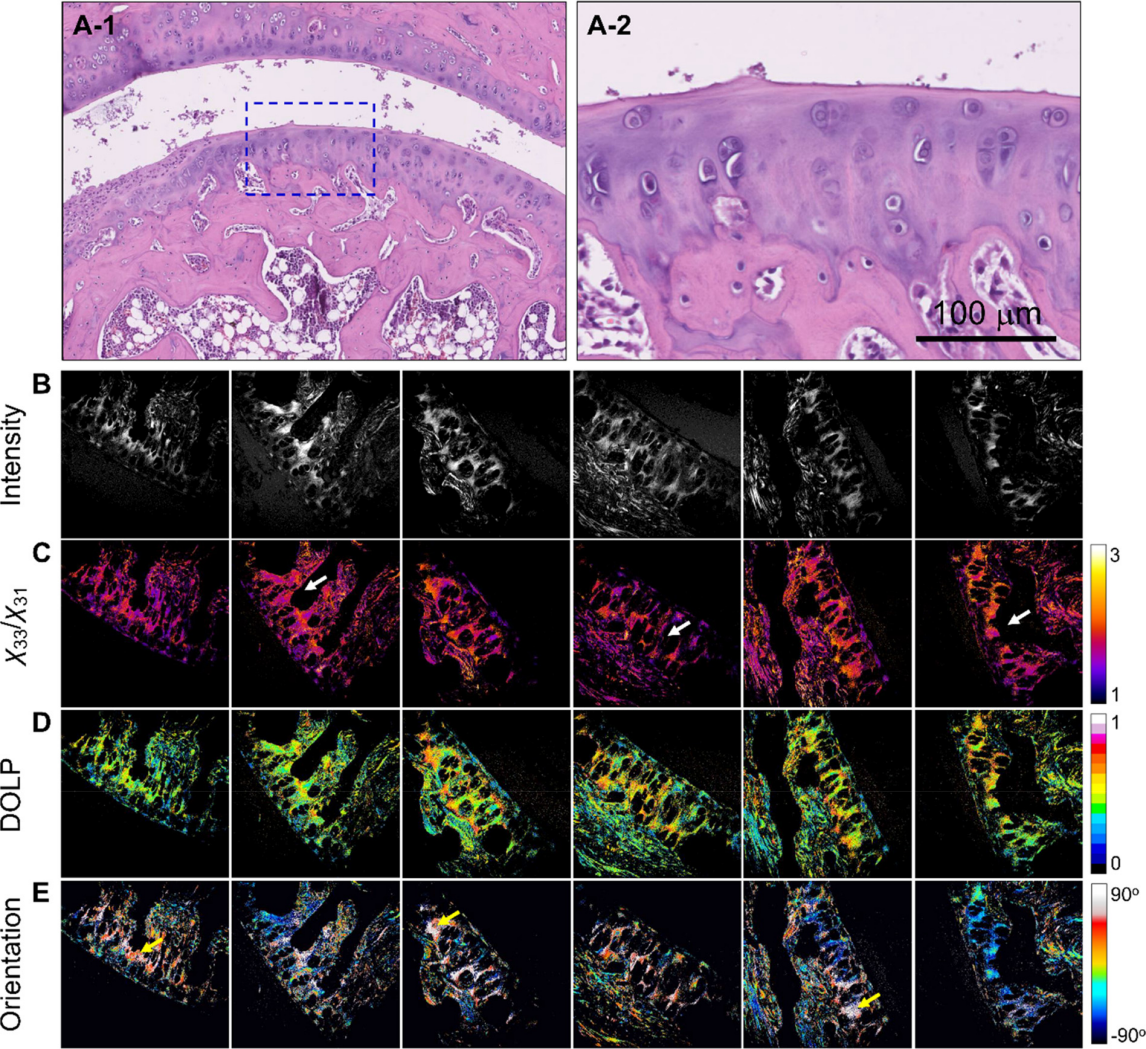
Images of RA tissue: (a-1) and (a-2) H&E-stained images, where (a-2) shows a magnified view of the region indicated in (a-1). (b)–(e) are the SHG intensity, *χ*_33_/*χ*_31_, DOLP, and planar orientation of the RA tissues, respectively. The white arrows indicate the erosion of cartilage and the swelling of chondrocyte areas, and the yellow arrows indicate areas with similar colors, showing nearly consistent fibril orientations due to the existence of type-I collagen. The color scale shows the values of each parameter. Image size = 400 × 400 *μ*m^2^. Images (b)–(e) are representative and were selected from the samples used (n = 5).

In the RA group [Figs. 5(a-1) and 5(a-2)], the H&E stained images revealed distinct morphological structures with cartilage erosion and chondrocyte swelling. SHG images [Fig. 5(b)] revealed an increased average *χ*_33_/*χ*_31_ value (n = 5) of 1.75 ± 0.337 [Fig. 5(c)] compared to that of RA-C, indicating altered tissue optical properties. Furthermore, an increase in DOLP (0.462 ± 0.192) was found in the RA group [Fig. 5(d)], which indicated that some of the cartilage areas that originally contained type-II collagen were transformed into type-I collagen. The orientation distribution in Fig. 5(e) indicates widespread collagen orientation, with transformed type-I collagen areas exhibiting a more consistent orientation (indicated by yellow arrows), a feature also observed in OA.

**FIG. 6.**
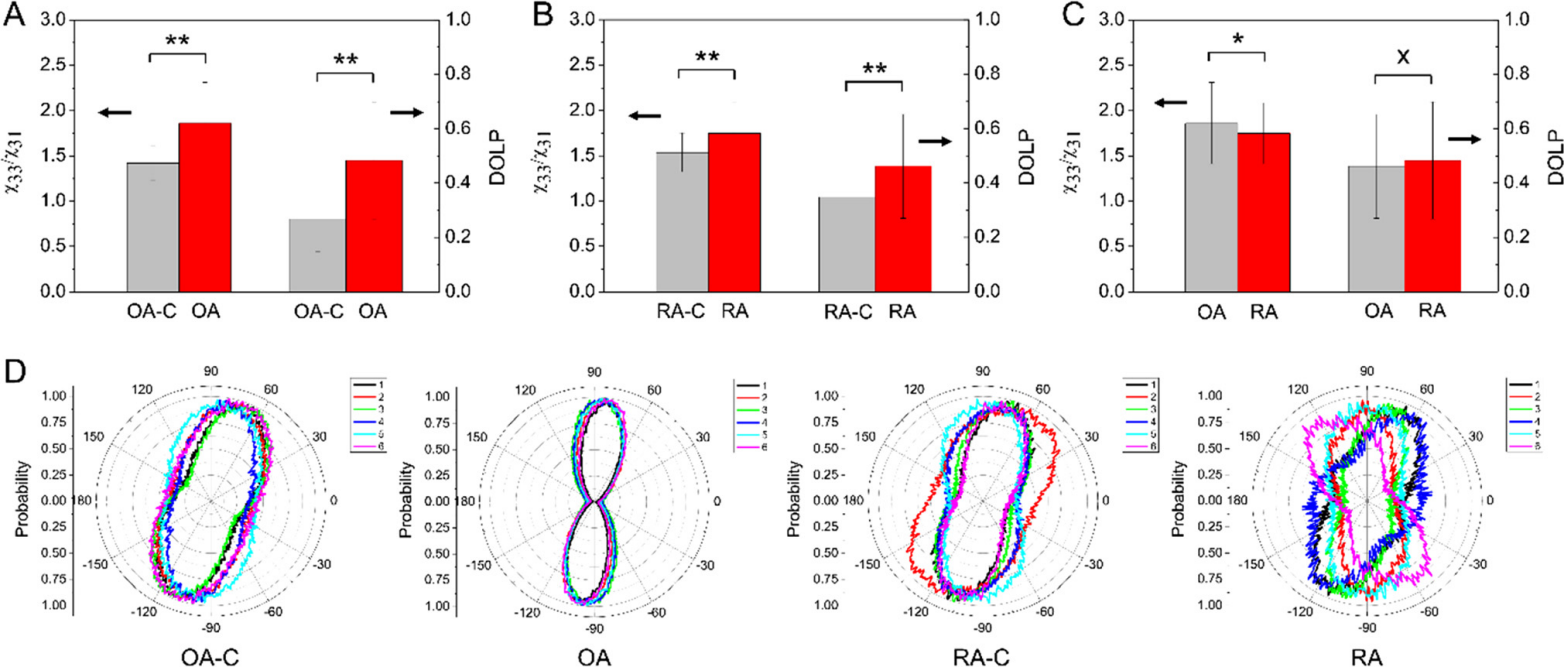
Histogram representation of the *χ*_33_/*χ*_31_ and DOLP features (n = 5) of the cartilage tissues: (a) OA-C vs OA, (b) RA-C vs RA, and (c) OA vs RA. ^*^ indicates *p* < 0.05 and ^**^ indicates *p* < 0.01. (d) Polar histogram of the planar orientation of collagen fibrils for OA-C, OA, RA-C, and RA. The numbers (1)–(6) shown in the top-right corner are the results corresponding to the images in Figs. 2(e), 3(e), 4(e), and 5(e) from left to right.

To highlight the structural variations between each condition, statistical analysis and subsequent comparative analysis were performed. Figures 6(a)–6(c) present the results for OA-C/OA, RA-C/RA, and OA/RA and one-way ANOVA tests, wherein the ^*^ symbols on histograms indicate statistical significance. Apart from the comparisons made between the control tissue and the respective pathologies of the cartilage, an additional comparison was made between the two pathologies, viz., OA and RA. The *χ*_33_/*χ*_31_ histogram in Fig. 6(c) highlights the different pathogenic mechanisms related to extracellular collagen degradation associated with OA and RA. The results of the orientation images are further represented as histograms, as shown in Fig. 6(d). The orientation distributions show a rough main orientation, except in the case of OA tissues, and are evenly spread across various orientation angles. It is possible to use the ratio between the maxima and minima probabilities at the respective angles. Thus, OA presented a higher ratio, indicating that the cartilage contains more oriented type-I collagen, which is consistent with the *χ*_33_/*χ*_31_ and DOLP results.

## III. DISCUSSION

Previous studies on OA and RA have introduced new imaging methods, which have been validated mostly through biological approaches.^26,27^ Although these methods offer novel avenues for understanding cartilage-related diseases, there is still a gap in the fundamental knowledge regarding the mechanism of collagen degradation. In this study, P-SHG imaging of cartilage tissue was performed, and statistical analyses of the structural parameters were included to validate this approach for investigating cartilage-related diseases.

Analysis of optical images is of paramount importance for drawing meaningful conclusions from the image data. Image textural analysis provides an overview of the changes or variations in the surface topology of the imaged tissue. Numerous applications of textural analysis of tissue structures have been reported.^28^ The textural parameters such as Contrast, Dissimilarity, Homogeneity, Energy, Correlation, and angular second moment (ASM) are known to provide significant details on the textural uniformity of tissue images. A high Contrast value for the tissue samples indicates larger differences between the pixel intensities than for a more complex structure. A similar inference is drawn from the feature Dissimilarity as well.^29^ In our results, the OA group showed higher Contrast and Dissimilarity than those of the control group, which is indicative of ECM distortion due to cartilage damage and the replacement of type-II collagen with more fibrillar type-I collagen, an observation typical of the progression of OA. However, in the case of RA, the results indicate greater complexity in the control tissue. This reduction in tissue complexity during disease could be attributed to the fact that cartilage mostly retains collagen structures in the ECM; however, these structures are progressively damaged due to inflammation and other enzymatic activities that occur during the progression of RA. The ASM, Correlation, Homogeneity, and Energy parameters of the GLCM textural features reflect the orderliness of textures, where higher values indicate uniformity in the gray-level distribution of the image.^30^ Furthermore, our results indicated that OA had lower values for the aforementioned features than those of the control and RA tissues. This is indicative of an increase in the complexity of cartilage tissue during the progression of OA, which is mostly due to the replacement of type-II collagen with more fibrillar type-I collagen by chondrocytes in the ECM.^6–9^

The morphology of cartilage and the behavior of chondrocytes can differ between OA and RA. These alterations can manifest in the structure of cartilage and in the metabolic processes of chondrocytes. According to relevant studies, chondrocyte viability and the metabolic state of cells, calculated from the signals of reduced nicotinamide adenine dinucleotide (NADH) and flavin adenine dinucleotide (FAD), are among the factors associated with disease progression and treatment efficacy.^21,31,32^ A shift in metabolic programming was observed in chondrocytes affected by degenerative arthritis by Liu *et al.*^33^ Importantly, the redox ratio and mitochondrial clustering were found to be lower in normal tissue than in OA tissue. In addition, a recent study^2^ revealed that during the onset and progression of OA, the cartilage ECM deteriorates as the composition changes from type-II to type-I collagen.^6–9^ In this way, cartilage lesions reduce the tensile strength that cartilage can withstand, resulting in irreversible changes.

Currently, there are a few assessments based on type-II collagen organization, as the size of collagen fibrils in cartilage is less than the diffraction-limited resolution provided by optical microscopy (i.e., sub micrometer scale), which makes it difficult to determine the molecular orientation of collagen fibrils without high-contrast images.^2^ However, the second-order susceptibility tensor^34–36^ and Stokes vector analysis^37,38^ used in P-SHG microscopy pave the way for overcoming the diffraction barrier and enable quantitative imaging of fibril orientation. In a recent study of P-SHG imaging of cartilage structure and its mechanical properties,^2^ the authors focused on variations in collagen fibril orientation and macroscopic organization to resist the imposed mechanical forces on the three zones in cartilage. However, there has been little discussion on the molecular organization of the ECM in diseased cartilage tissues using P-SHG imaging. To provide a better understanding of the collagen degradation processes observed in OA and RA, we used P-SHG imaging not only to observe the morphology of normal and diseased cartilage tissues but also to determine the molecular structural parameters. Further statistical and comparative analyses allowed the assessment and classification of cartilage tissues based on their pathology. Importantly, the different pathological mechanisms determined by our experiments revealed that P-SHG imaging is capable of investigating the multiscale structures of cartilage and is well suited for clinical diagnosis due to its simple image calculation and possibility of label-free detection.

The measured *χ*_33_/*χ*_31_ values of normal cartilage tissues were 1.42 ± 0.195 and 1.537 ± 0.216 for OA-C and RA-C, respectively, which are reflective of pure type-II collagen and are consistent with the results of the reference.^2,39^ The wide range of values can be attributed to the effect of the molecular tilt angle because it varies with different zones of cartilage and may be due to imperfect tissue dissection.^34,40^ In contrast, the *χ*_33_/*χ*_31_ values of OA tissues were 1.861 ± 0.449 and those of RA tissues were 1.75 ± 0.337. The higher values are because of the partial transition of collagen fibrils from type-II to type-I, resulting in modulations between collagen types that exhibit *χ*_33_/*χ*_31_ values between those of normal type-I and type-II collagen. In addition, the correlation between DOLP and the degree of tissue birefringence by collagen fibrils depends on fibril orientation and organization. To ease the complexity of image analysis, the *χ*_33_/*χ*_31_ values were linked with DOLP values using the same polarization images for analysis, and the results were found to be linearly dependent. In contrast to PLM images, P-SHG images have a comparatively lower depolarization effect on the SHG photons and are sensitive to the fibril orientation. We postulate that the birefringence of normal tissues is lower than that of diseased tissues because of the transition of type-I collagen in cartilage. However, regarding the degree to which the DOLP increases from normal to diseased tissues and the higher the DOLP for OA tissue than for RA tissue, the degradation mechanism of collagen fibrils in OA is dominated by the transition of collagen types, while that in RA tissue is influenced mainly by the erosion and swelling of collagen fibrils and partly by the collagen type transition. In the previous discussion,^2^ collagen fibril remodeling was the degradation mechanism of OA, which is equivalent to our finding that collagen type transition is the mechanism of collagen fibril remodeling and reorientation. Furthermore, according to the polar histograms in Fig. 6(d), OA clearly has a more consistent fibril orientation than the other samples, highlighting the characteristics of type-I collagen. Thus, the ratio between the maxima and minima probability at the respective angle could be a useful factor for defining the modulation between type-I and type-II collagen.

Furthermore, previous studies have investigated tissue structures using P-SHG microscopy and have shown that it is an effective tool for studying collagen-rich tissues.^41–43^ He *et al.* highlighted the application of SHG microscopy for the visualization of articular cartilage. The authors discuss the characteristics of SHG microscopy, which makes it an ideal tool for visualizing collagen fibrils, thus enabling an understanding of the relationship between collagen and other components of the ECM.^44^ Wu *et al.* studied the collagen fibril matrix of Achilles tendons derived from rabbits. SHG microscopy was used for visualization of the tissue specimens, and the ImageJ software and its plugins were used for image analysis. The findings of the study suggest that type-I collagen is predominantly present in tendons. The authors further suggest the application of the image analysis technique during tissue engineering for restoring the collagen structure of a tendon without tissue staining.^45^ Further studies were also performed to analyze the structure of the articular cartilage. Mansfield *et al.* utilized P-SHG to analyze collagen fibril organization in human articular cartilage and found that the technique revealed regional and local patterns in the articular cartilage and its response to changes in mechanical loading.^2^ Furthermore, Kumar *et al.* applied P-SHG to evaluate articular cartilage degradation and reported that this technique could reveal changes in the molecular structure of the cartilage matrix associated with degeneration. This study also revealed the potential of P-SHG microscopy to monitor the progression of OA.^46^

In addition to the above-mentioned discussion of type-I and type-II collagen present in the ECM of OA and RA tissues, P-SHG microscopy has shown great promise for investigating collagen-related diseases, such as liver fibrosis and keloid scarring.^47,48^ With the ability to distinguish between different collagen types and organization patterns of the ECM followed by specific diseases or degradation processes, P-SHG can be used to study cartilage fibrosis by measuring the structural factors indicated in this work for the phenomenon of cartilage fracture and the deposition of type-I and type-III collagens.^49^ Furthermore, as OA is multifactorial with diverse ECM structures involving unique collagen organization and remodeling, our work provides a good example in which the high structural specificity based on the measured structural factors could be used to assess and distinguish between different types of OA, i.e., primary and secondary OA, and arthrofibrosis involving excessive scar tissue formation within the joint and/or the tissues around it.^50^

Our study builds upon previous research by using textural analysis to extract quantitative information from P-SHG images, which enhances the understanding of tissue characteristics during disease progression. This study compares cartilage samples from normal, OA, and RA tissues and reveals distinct parameters in the cartilage samples, reflecting variations in collagen fibril arrangement and organization across different pathological states. The imaging technique, the quantitative image analysis, and the parameters used in this study have great potential for disease diagnostics, fostering a clear understanding of collagen fibril degradation in cartilage. We also postulate the applicability of the techniques used in this study to evaluate the effectiveness of treatments aimed at modifying the collagen matrix in cartilage and arthrofibrosis, a condition characterized by excessive scar tissue formation in joints, leading to restricted movement and pain.^50^ By monitoring changes in collagen organization, density, and alignment, the response to treatment can be assessed over time. Furthermore, this technique can be used to monitor the regeneration of cartilage tissue after injury or surgery, providing insight into the quality and organization of the newly formed collagen matrix and assisting in the assessment of tissue healing and repair processes.

## IV. CONCLUSIONS

In conclusion, P-SHG microscopy has emerged as a potent diagnostic tool for OA, RA, and other collagen-related diseases. It significantly enhances molecular contrast, facilitating the differentiation of normal tissues from those affected by OA and RA. The derived parameters, including *χ*_33_/*χ*_31_, DOLP, and the collagen fibril orientation distribution, offer valuable metrics for disease diagnosis and classification. This technique provides insight into the collagen fibril degradation process in cartilage and holds the potential to increase diagnostic accuracy, enabling personalized medicine, early intervention, and improved patient outcomes.

## V. METHODS

### A. Tissue sample preparation

The animal experiments complied with ethical standards under approval number CMUIACUC-2022-185 from the China Medical University Committee for the Use and Care of Animals. For the OA study, 12-week-old male Sprague-Dawley (SD) rats (BioLASCO Taiwan Co., Ltd., Taipei, Taiwan) were utilized. The rats were reared in an animal facility for at least 1 week for stabilization before enzyme injection.^51^ Standard conditions were maintained for the rats, with five in each control and diseased group.

The rats were anesthetized with 2.5% isoflurane (Abbott, USA) at a 70 ml/min flow rate before every injection. OA was induced in the right knee through the enzymatic injection of 0.2 ml of 4% papain solution (Sigma-Aldrich, USA) with 0.1 ml of 0.03 M cysteine (Sigma-Aldrich, USA) as an activator, while medical saline was injected into the left knee as a control. The injection was repeated on the fourth day and the seventh day of treatment. Two weeks after the last injection, the rats were sacrificed by an overdose of carbon dioxide, and the knee cartilage was extracted for histological analysis and P-SHG imaging.^52^ The extracted tissues were fixed with 4% paraformaldehyde (PFA) in PBS, decalcified in 0.5 M EDTA for 2 weeks, embedded in paraffin, and serially sectioned at 5 *μ*m thickness in the sagittal direction.

In the study of RA, male SD rats aged 12 weeks were used, with five rats in each control and diseased group. Collagen-induced arthritis (CIA) was induced by intradermal injection of type-II collagen (from bovine nasal, Elastin Products Company, Inc.) dissolved in 0.1 M acetic acid and mixed with complete Freund's adjuvant (CFA) at a 1:1 ratio for collagen emulsion preparation. After 7 days, a booster dose of the initial injection was administered in a similar manner to the rats. The rats were monitored daily for arthritis. Notably, inducing RA typically requires at least 2 weeks to observe pathological changes. Each rat exhibited erythema and/or swelling in the limbs at the time of sacrifice on day 27 after immunization with type-II collagen, followed by a 3-week decalcification process before histological sectioning. Then, the biopsy samples were fixed in 10% (wt./vol.) buffered formalin, embedded in paraffin, longitudinally sectioned, and covered with a cover glass for P-SHG imaging.

### B. P-SHG microscopy

A custom-built nonlinear laser scanning microscope was used to obtain SHG images, as described in the literature.^34,35^ The pump source was a 1030-nm fiber laser system (Kasmoro-1030, mRadian, Taiwan) with the following parameters: 48 MHz pulse repetition rate, ∼100 fs pulse width, and ∼3 W total power. Before polarization-resolved imaging, the polarization state of the laser beam was tailored by a polarization kit composed of a polarizer (LPNIRE100-B, Thorlabs, USA), a half-wave plate (AHWP05M-980, Thorlabs, USA), and a quarter-wave plate (AQWP05M-980, Thorlabs, USA) for producing linear polarization (LP) at 0°, 45°, 90°, and 135° relative to the x-axis. The beam was deflected by a 2D galvo-mirror assembly (GVS012/M, Thorlabs, USA) combined with an optical 4-f system to relay the beam. The beam passed through the system and was focused by an objective lens (UPlanSAPO 20x/0.75, Olympus, Japan). With samples as tissue slides (<10 *μ*m thickness), the deterioration in the polarization of the SHG signals in the forward direction was negligible. The laser power at the focal spot was 25 mW, which further reduced the probability of photodamage on the sample. For forward detection, a condenser lens (ACL25416U-A, Thorlabs, USA) was used for photon collection. The photons were filtered through a colored glass filter (FGB37M, Thorlabs, USA) and an interference filter (FB520-10, Thorlabs, USA) before they reached the photomultiplier tubes (R3896, Hamamatsu, Japan). The pure SHG photons were then detected and digitized to form images. Image analyses and data presentation were conducted using ImageJ/Fiji and Origin 2022 software, respectively.

### C. Image preprocessing and feature extraction

Images were organized into folders for efficient analysis, converted to 8-bit grayscale, and subjected to textural analysis using the GLCM method. Key textural features—Contrast, Dissimilarity, Correlation, angular second moment (ASM), Homogeneity, and Energy—were extracted through the GLCM algorithm. The GLCM is constructed by iterating through the image and counting the number of occurrences of a gray level adjacent to another gray-level pixel, i.e., a pair of pixels (i, j), at a specified pixel distance. The matrix components indicate the likelihood of gray-level co-occurrence between the pixels, wherein the matrix rows and columns represent the gray levels in the image. Parameters were estimated based on average values for four orientations (horizontal, vertical, and diagonal). Contrast is a measure of the local variations in an image, while Homogeneity measures the density distribution of the elements in the co-occurrence matrix. ASM is a measure of the textural uniformity of the image, wherein a higher maximum value of unity corresponds to high textural and structural uniformity and is, thus, indicative of the orientation of the collagen fibrils in an SHG image. Correlation measures the linear dependency of the gray levels on the neighboring pixels. Energy and Dissimilarity also measure the uniformity of an image.^30,53^

### D. Theory for the derivation of the susceptibility tensor ratio (*χ*_33_/*χ*_31_), degree of linear polarization (DOLP), and planar orientation

#### 1. Susceptibility tensor ratio, *χ*_33_/*χ*_31_

SHG is a second-order nonlinear optical process and can be formulated with a structural tensor *χ*^(2)^, while the corresponding SHG intensity is described by multiplying *χ*^(2)^ and the imposed electric field.^54–58^ By manipulating the electric field (i.e., polarization state), the dependence of the SHG intensity on the varying polarization states is shown in each pixel of the images, where structural information, such as the local arrangement of SHG-active molecules within the supramolecule, intrinsic optical nonlinearities, and helical pitch angle of SHG-active molecules, is obtained through *χ*^(2)^ tensor analysis. A change in the *χ*_33_/*χ*_31_ ratio was found between normal and malignant melanoma skin tissues.^59^ This value was found to be closely related to the molecular structure and mechanical tension of the collagen fibrils,^60^ making it a promising parameter for differentiating normal from diseased cartilage tissues. To streamline the overall processes, SHG images at four polarizations were utilized here to derive the factor of *χ*_33_/*χ*_31_. The SHG intensity was assumed to be a function of the polarization direction *φ_n_* and molecular orientation *θ*,^59,61^ written as $I_{n}\left(\theta \right)=I_{n}\left(\theta ,\;\varphi_{n}\right)$. *n* denotes the *n*th polarization state in the direction of *nπ*/4. To compute *χ*_33_/*χ*_31_, two combined equations are needed, as shown below:

$$\begin{array}{c} I_{02}\left(\theta \right)=\frac{\left[I_{0}\left(\theta \right){-I}_{2}\left(\theta \right)\right]}{2},\\ I_{31}\left(\theta \right)=\frac{\left[I_{3}\left(\theta \right){-I}_{1}\left(\theta \right)\right]}{2}. \end{array}$$ (1)

The amplitude of the polarization contrast $\left|V\right|$ equals

$$\left|V\right|=\sqrt{I_{02}^{2}+I_{31}^{2}},$$ (2)

where *U* is the average intensity over the four polarization SHG images and is given by

$$U=\left(I_{0}\left(\theta \right)+I_{1}\left(\theta \right)+I_{2}\left(\theta \right)+I_{3}\left(\theta \right)\right)/4.$$ (3)

Consequently, the ratio of $\left|V\right|/U$ is used to determine *χ*_33_/*χ*_31_ according to Eq. (4) by presuming that *χ*_33_/*χ*_31_ > 0

$$\frac{\chi_{33}}{\chi_{31}}=\frac{\frac{\left|V\right|}{U}+2\sqrt{4\left((\left|V\right|/U\right)+1)-5{\left(\left|V\right|/U\right)}^{2}}}{4-3\left(\left|V\right|/U\right)}.$$ (4)

#### 2. Degree of linear polarization (DOLP)

DOLP is a parameter determining the fraction of fully linearly polarized light and is linked to the fibrillar arrangement parallel to the linear polarization state, the integrity of the ultrastructure of the SHG-active molecules, and tissue birefringence.^38,62,63^ The formula is shown in Eq. (5), wherein *S*_0_, *S*_1_, and *S*_2_ are Stoke vectors and can be represented by *I*_0_ + *I*_2_, *I*_0_ − *I*_2_, and *I*_1_ − *I*_3_, respectively. Notably, the definition of the measured SHG intensity, *I*, with a subscripted number is the same as that previously indicated.^38^ The range of DOLP is from 0 to 1, where 1 represents fully linearly polarized light and 0 represents depolarized light. This imaging scheme can be easily adopted to measure the aforementioned factors to calculate DOLP

$$\mathrm{DOLP}=\frac{\sqrt{{S_{1}}^{2}+{S_{2}}^{2}}}{S_{0}}.$$ (5)

#### 3. Derivation of the planar orientation

Like *χ*_33_/*χ*_31_, the planar orientation was determined using the theory of orientation field SHG.^59,61^ This method required only P-SHG images with linear polarizations at 0°, 45°, 90°, and 135° for analysis, providing a quick overview of the molecular orientation distribution in the tissue sample. The formula used to determine the planar orientation is shown in the following equation:

$$\theta_{0}={\text{tan}}^{-1}\frac{I_{3}-I_{1}}{I_{0}-I_{2}}.$$ (6)

The factors in Eq. (6) are analogous to those in Eq. (1). Therefore, our imaging approach provides an alternative for determining planar orientation through image calculations, eliminating the need for intricate data fitting based on an analytical model.^34,54,56^

### E. Statistical analysis

For each condition (normal mouse articular cartilage as a control, OA, and RA), five samples were statistically analyzed. The ImageJ software was used to calculate the various polarization parameters from the whole images, after which the average value and standard deviation were calculated. To demonstrate the structural specificity for diseased tissue classifications, one-way ANOVA test was performed on the normal tissue, OA, and RA data to assess the statistical significance (p < 0.05) of the datasets. Additionally, given the importance of the collagen fibril orientation distribution in defining pathological states, the calculated planar orientation results were used to generate a polar histogram for subsequent statistical analysis.

## SUPPLEMENTARY MATERIAL

See the supplementary material for the datasets of P-SHG images of OA-C, OA, RA-C, RA, and bone tissues and the subsequent image analyses.

## Data Availability

The data that support the findings of this study are available from the corresponding author upon reasonable request.
